# Neuropsychiatric involvement in juvenile-onset systemic lupus erythematosus: Data from the UK Juvenile-onset systemic lupus erythematosus cohort study

**DOI:** 10.1177/09612033211045050

**Published:** 2021-10-02

**Authors:** Teresa Giani, Eve MD Smith, Eslam Al-Abadi, Kate Armon, Kathryn Bailey, Coziana Ciurtin, Joyce Davidson, Janet Gardner-Medwin, Kirsty Haslam, Dan P Hawley, Alice Leahy, Valentina Leone, Flora McErlane, Devesh Mewar, Gita Modgil, Robert Moots, Clarissa Pilkington, Francesca Pregnolato, Athimalaipet V Ramanan, Satyapal Rangaraj, Phil Riley, Arani Sridhar, Nick Wilkinson, Rolando Cimaz, Michael W Beresford, Christian M Hedrich, on behalf of the UK JSLE Cohort Study

**Affiliations:** 1 Rheumatology Unit, AOU Meyer, Florence, Italy; 2 Department of Medical Biotechnology, University of Siena, Siena, Italy; 3 Department of Women’s & Children’s Health, Institute of Life Course and Medical Sciences, University of Liverpool, Liverpool, UK; 4 Department of Paediatric Rheumatology, 4593 Alder Hey Children’s NHS Foundation Trust Hospital, Liverpool, UK; 5 Department of Rheumatology, 22078 Birmingham Children’s Hospital, Birmingham, UK; 6 Department of Paediatric Rheumatology, 2153 Cambridge University Hospitals, Cambridge, UK; 7 Department of Paediatric Rheumatology, 6397 Oxford University Hospitals NHS Foundation Trust, Oxford, UK; 8 Centre for Adolescent Rheumatology, 308348 University College London, London, UK; 9 Department of Paediatric Rheumatology, 59841 Royal Hospital for Sick Children, Edinburgh, UK; 10 Department of Child Health, 3526 University of Glasgow, Glasgow, UK; 11 Department of Paediatrics, 111991 Bradford Royal Infirmary, Bradford, UK; 12 Department of Paediatric Rheumatology, 7316 Sheffield Children’s Hospital, Sheffield, UK; 13 Department of Paediatric Rheumatology, 105634 Southampton General Hospital, Southampton, UK; 14 Department of Paediatric Rheumatology, 4472 Leeds Children Hospital, Leeds, UK; 15 Paediatric Rheumatology, Great North Children’s Hospital, Royal Victoria Infirmary, 105563 Institute of Cellular Medicine, Newcastle University, Newcastle Upon Tyne, UK; 16 Department of Rheumatology, 159020 Royal Liverpool University Hospital, Liverpool, UK; 17 Department of Paediatrics, 156775 Musgrove Park Hospital, Taunton, UK; 18 Department of Rheumatology, 89542 University Hospital Aintree, Liverpool, UK; 19 Department of Paediatric Rheumatology, 4956 Great Ormond Street Hospital, London, UK; 20 Immunorheumatology Research Laboratory, Auxologico Institute, Milan, Italy; 21 University Hospitals Bristol NHS Foundation Trust & Bristol Medical School, 156596 University of Bristol, Bristol, UK; 22 Department of Paediatric Rheumatology, 9820 Nottingham University Hospitals, Nottingham, UK; 23 Department of Paediatric Rheumatology, 105554 Royal Manchester Children’s Hospital, Manchester, UK; 24 Department of Paediatrics, 156756 Leicester Royal Infirmary, Leicester, UK; 25 Guy’s & St Thomas’s NHS Foundation Trust, 443490 Evelina Children’s Hospital, London, UK; 26 ASST Gaetano 4591 Pini-CTO, Milan, Italy.; 27 Department of Clinical Sciences and Community Health, Research Center for Adult and Pediatric Rheumatic Diseases, University of Milan, Milan, Italy

**Keywords:** Systemic lupus erythematosus, neuropsychiatric, central nervous system, peripheral nervous system, pediatric, juvenile

## Abstract

**Introduction:**

Juvenile-onset systemic lupus erythematosus (JSLE) is a rare autoimmune/inflammatory disease with significant morbidity and mortality. Neuropsychiatric (NP) involvement is a severe complication, encompassing a heterogeneous range of neurological and psychiatric manifestations.

**Methods:**

Demographic, clinical, and laboratory features of NP-SLE were assessed in participants of the UK JSLE Cohort Study, and compared to patients in the same cohort without NP manifestations.

**Results:**

A total of 428 JSLE patients were included in this study, 25% of which exhibited NP features, half of them at first visit. Most common neurological symptoms among NP-JSLE patients included headaches (78.5%), mood disorders (48.6%), cognitive impairment (42%), anxiety (23.3%), seizures (19.6%), movement disorders (17.7%), and cerebrovascular disease (14.9%). Peripheral nervous system involvement was recorded in 7% of NP-SLE patients. NP-JSLE patients more frequently exhibited thrombocytopenia (<100 × 10^9^/L) (*p* = 0.04), higher C-reactive protein levels (*p* = 0.01), higher global pBILAG score at first visit (*p* < 0.001), and higher SLICC damage index score at first (*p* = 0.02) and last (*p* < 0.001) visit when compared to JSLE patients without NP involvement.

**Conclusions:**

A significant proportion of JSLE patients experience NP involvement (25%). Juvenile-onset NP-SLE most commonly affects the CNS and is associated with increased overall disease activity and damage.

## Introduction

Systemic lupus erythematosus (SLE) is a severe and potentially life-threatening chronic autoimmune/inflammatory disease that can affect any organ system. The molecular pathophysiology of SLE is complex and incompletely understood. While relatively few SLE patients experience “purely” genetic forms that are caused by mutations in single genes, most individuals exhibit genetic predispositions in the context of environmental and hormonal factors.^1^ The key contribution of genetic factors is underscored by highly variable incidences between ethnicities.^2^ The highest incidence and prevalence of SLE worldwide have been reported in North America [23.2/100 000 person-years (95% CI: 23.4–24.0) and 241/100 000 people (95% CI: 130–352), respectively], while lowest incidences were reported in Africa and Ukraine (0.3/100 000 person-years).^3^ In the UK, the incidence is reported to range around 4.91 cases/100,000 person-years.^4^

Approximately 15–20% of SLE patients develop disease before their 16th birthday and are diagnosed with juvenile-onset systemic lupus erythematosus (JSLE).^1,5^ Disease onset in childhood and adolescence is associated with more severe disease presentations, increased organ damage (already at the time of diagnosis), and an even more variable clinical and serological picture when compared to the adult age group.^6^

Neuropsychiatric (NP) involvement is a potentially severe complication that can increase disease burden, and cause significant damage and disability.^7^ The variable presentation of NP disease is reflected by the presence of 19 items in the American College of Rheumatology (ACR) classification for NP-SLE, including items related to both the central (CNS) and/or peripheral (PNS) nervous system.^8^ Though based on a limited number of reports, NP-SLE appears to be more common and aggressive among children and adolescents as compared to patients with adult-onset SLE.^9–11^ As many as 22–95% of JSLE patients develop NP disease,^12^ while 14–80% of adult-onset SLE patients are affected.^13^ To date, few comparative studies have analyzed NP involvement in children and adults. Reports underscore that neurologic manifestations are not uncommon in JSLE, contributing to the increased morbidity and mortality demonstrated in the pediatric age group.^10,14–16^ Thus, timely diagnosis, and treatment of NP-SLE is critical to improve patients’ quality of life and prevent damage.^14^

This study aimed to investigate the prevalence, demographic characteristics, and clinical features of NP disease in JSLE patients enrolled in the UK JSLE Cohort Study. Furthermore, associations between NP involvement and other clinical and/or laboratory features, as well correlation with disease severity and outcomes were investigated.

## Materials and Methods

### Study Cohort

This study was based upon the UK JSLE Cohort, a multidisciplinary, multicenter collaborative network established in 2006 with the primary aim of determining the clinical characteristics of JSLE patients across the UK, as well as supporting a program of clinical translational research (for details, see http://www.liv.ac.uk/ukjsle). The Study collects detailed data on demographics, ACR criteria for the classification of SLE, disease activity, medication use, and disease damage scores on a regular basis (see below). The UK JSLE Cohort is managed by the national coordinating center in Liverpool (CI: Beresford), with participating institutions including the majority of pediatric rheumatology/nephrology centers in the UK (*n* = 23). The JSLE Cohort Study has full ethical approvals in place (National Research Ethics Service North West, Liverpool, UK, reference 06/Q1502/77), and patient/parental consent or assent to take part in the study was obtained from all families. The research was carried out in accordance with the Declaration of Helsinki.

#### Patients

All JSLE patients were followed between August 2006 and August 2019. Patients were aged ≤16 years at the time of diagnosis and satisfied ≥4 ACR classification criteria for SLE.^17^ Patients with NP manifestations preceding the diagnosis of JSLE by more than 6 months were not included in the analysis. Patients with isolated headaches, mood or anxiety disorders as the only neurological manifestation were excluded from the group of NP-JSLE patients, as these symptoms are relatively prevalent in the general population, and therefore may be unrelated to JSLE. However, the importance of these symptoms was considered within the “no NP involvement” sub-cohort of the study.

#### Data collected

Demographic (gender, age at diagnosis, ethnicity, and family history for autoimmune disease), clinical and laboratory information (ACR-1997 classification criteria for SLE, the Systemic Lupus International Collaborating Clinic Damage Index (SLICC-SDI),^18,19^ the pediatric version of British Isles Lupus Assessment Group (BILAG; pBILAG2004) disease activity score,^18^ laboratory values including autoantibody status of JSLE patients enrolled in the UK JSLE Cohort Study were accessed. Neurological manifestations were classified using the standardized nomenclature and case definitions for the 19 NP manifestations linked to SLE developed in 1999 by the ACR ad hoc Committee.^8^ On the basis of the ACR glossary, which provides an exhaustive index of other potential causes for each of the NP manifestations, NP events which were not deemed to be SLE-related were not reported in the NP-SLE specific fields of the database.

#### Statistical analysis

For comparative analyses, patients were grouped on the basis of SLE in the presence or absence of NP involvement. Furthermore, JSLE patients with NP involvement were subgrouped into “early” appearance of NP symptoms, if present already at first visit, or “late” NP involvement if it developed later, and was registered at last visit. Lupus‐related variables that were assessed included the number of ACR classification criteria present at first and last visit,^17^ the pBILAG2004 disease activity scores at first and last visit,^18^ and the SLICC-SDI at first and last visit.^18,19^ “Severe” NP involvement was defined by a pBILAG score of A in the NP domain, “moderate” NP involvement as a B, “mild” NP involvement as C; NP pBILAG scores of D reflect a history of NP involvement and current inactivity, E the absence of NP involvement.

Descriptive statistical analysis (mean, SD) was performed using Microsoft Excel 2016. Comparison between groups was performed using Mann–Whitney U or Chi-square tests, as appropriate (Minitab19.2020.1; GraphPadPrism 6.0). Due to the exploratory nature of the analysis, adjustments for multiple comparisons were not made. Statistical significance was assumed at *p* < 0.05.

## Results

### Patient demographics

A total of 428 JSLE patients recruited to the UK JSLE Cohort Study were included in this study. One hundred and seven (25%) developed NP features, with onset early in the disease (already present at first visit) in 52/107 (48.5%) of them. The mean follow-up period was 4.8 years (range: 0.2–15.4) for the entire cohort, and 5.6 years (range: 0.2–14.4) for patients with NP involvement (*p* = 0.04). Demographic information and family history of the study population are summarized in Table 1. No significant differences emerged in relation to age at disease onset and/or diagnosis, ethnicity, gender, and family history for autoimmune diseases when comparing subjects with and without NP involvement.

**Table 1.** Patient demographics, family history, and neuropsychiatric medical history.

	Total JSLE patients *n* = 428	NP involvement *n* = 107<	No NP involvement *n* = 321	*p value*	Early NP involvement *n* = 52	Late NP involvement *n* = 55	*p value*
Age at diagnosis (mean) in years (range)	12.2 (0.1–17.9)	12 (0.7–17.9)	12.2 (0.1–17)	0.64	11.9 (2.5–16.8)	12.1 (0.7–17.9)	0.71
F:M ratio	5.4:1	5.2:1	5.5:1	0.87	4.7:1	5.8:1	0.69
Ethnicity				0.90			0.53
White Caucasian	220 (51.4%)	57 (53.2%)	163 (50.7%)		25 (48%)	32 (58.1%)	
Black							
African	37 (8.6%)	7 (6.5%)	30 (9.3%)		5 (9.6%)	2 (3.6%)	
Black Caribbean	35 (8.1%)	10 (9.3%)	25 (7.7%)		4 (7.6%)	6 (10.9%)	
East Asian	28 (6.5%)	7 (6.5%)	21 (6.5%)		3 (5.7%)	4 (7.2%)	
South Asian	100 (23.3%)	26 (24.2%)	74 (23%)		15 (28.8%)	11 (20%)	
Family history of AID							
Any AID	207 (48.3%)	45 (42%)	162 (50.4%)	1	22 (42.3%)	29 (52.7%)	1
SLE	52 (12.1%)	10 (9.3%)	42 (13%)	0.3	3 (5.7%)	7 (12.7%)	0.22
CTD	8 (1.8%)	3 (2.8%)	5 (1.5%)	0.41	2 (3.8%)	1 (1.8%)	0.53
Thyroid	50 (11.6%)	10 (9.3%)	40 (12.4%)	0.38	5 (9.6%)	5 (9%)	0.92
IDDM	34 (7.9%)	9 (8.4%)	25 (7.7%)	0.83	6 (11.5%)	4 (7.2%)	0.67
Rheumatoid Arthritis	63 (14.7%)	13 (12.1%)	50 (15.5%)	0.38	6 (11.5%)	7 (12.7%9	0.85

“Early” neuropsychiatric juvenile systemic lupus erythematosus (NP-JSLE) patients had NP features at the first recorded visit. AID, Autoimmune disease; CTD, Connective Tissue Disease; IDDM, Insulin-Dependent Diabetes Mellitus. For continuous variables, Mann–Whitney *U* test was used; for categorical variables, Chi-square test was used.

### Clinical characteristics of NP-SLE

**Figure 1.**
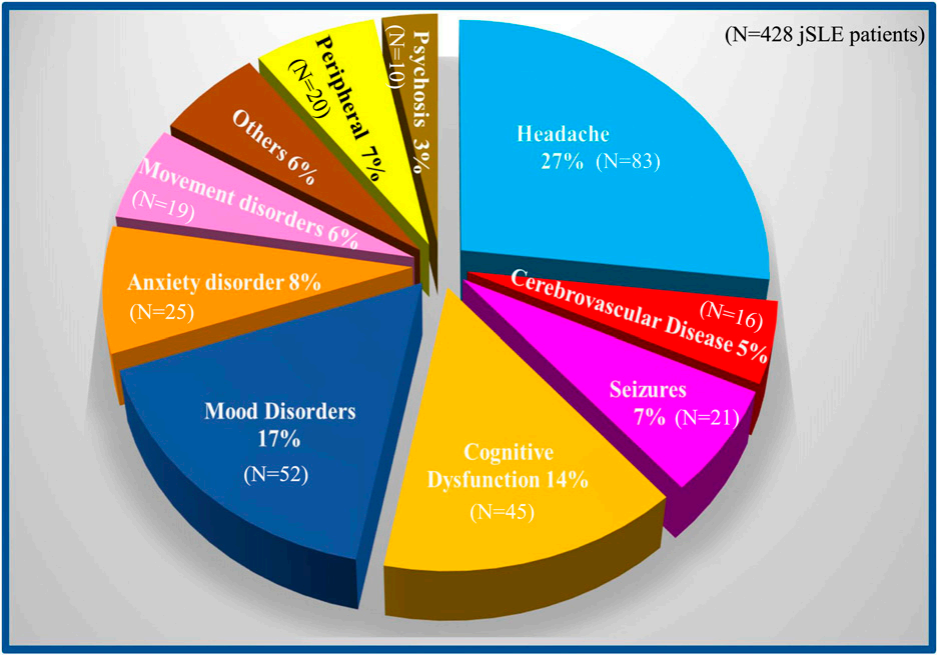
Frequency of neuropsychiatric syndromes. Distribution of each neuropsychiatric syndrome in the present series among the 19 standardized neuropsychiatric syndromes linked to systemic lupus erythematosus identified by the American College of Rheumatology ad hoc Committee.^8^ Peripheral, neuropsychiatric feature involving the peripheral nervous system. “Others” include: acute confusional state, aseptic meningitis, Guillain Barrè syndrome, and myelopathy that individually represent less than 2.5% of all manifestations.

The majority of NP manifestations (92.7%) were related to involvement of the CNS, as opposed to the PNS (Figure 1). Even after the exclusion of isolated headaches (see Materials & Methods), headaches were the most frequently observed potential symptom of NP-SLE, observed in 84/107 (78.5%) patients. Tension headaches represented 75% of all headache types (63/84 of patients), followed by episodic headaches (38/84 patients), “severe” headaches (23.8%, 20/84 patients), cluster headaches (3.5%, 3/84 patients), and hypertension-related headaches (1.2%, 1/84 patient). Fifty-four patients (64%) experienced more than one type of headache. Among the 321 JSLE patients with “no NP involvement” sub-cohort, headaches were reported by 177/321 (55.1%), with tension headaches being the most common (55%), while “severe” headaches were reported by 5% of these, and 16/177 (9%) experience at least of two different co-existing types of headaches.

Mood disorder was the second most common NP manifestation, documented in 52/107 (48.6%) NP-SLE patients (9 of whom had major depression), followed by cognitive dysfunction (45/107, 42%), anxiety disorder (25/107, 23.3%), seizures (21/107, 19.6%), movement disorders (19/107, 17.7%), cerebrovascular disease (16/107, 14.9%), mononeuropathy (9/107, 8.4%) acute confusional state (7/107, 6.5%), psychosis (10/107, 9.3%), and cranial neuropathy (6/107, 5.6%). Table 2 summarizes the prevalence of each NP manifestation in the studied cohort, also offering a comparison with comparable pediatric series published within the last 15 years.

**Table 2.** Comparison with other studies on juvenile-onset systemic lupus erythematosus (JSLE) with a neuropsychiatric (NP) involvement. SD, standard deviation; NS, nervous system.

	Present study	YU et al.^32^	Khajezadeh et al.^33^	Zambrano et al.^35^	Singh et al.^34^
Country of study	UK	China	Iran	Colombia	India
Cohort size • Total JSLE patients, N • NP-SLE patients, N (%)	428 107 (25%)	185 64 (34.6%)	146 41 (28%)	90 30 (33.3%)	53 27 (50.9%)
F:M ratio	5.4:1	5.9:1	3:1	5.4:1	2.8:1
Mean age (SD.)	12.2 (±3.1)	13.2 (±3	10.2 (±3)	12.2	9.9 (±3.2)
Distribution of NP features (%) among NP-JSLE patients					
Central NS Peripheral NS	93 7	97 3	99.3 0.7	87 13	94.4 5.6
Aseptic meningitis	1.8	0	0.7	NA	3.7
Acute confusional state	6.5	12.5	NA	NA	7.5
Anxiety disorder	23.3	6.3	NA	NA	1.8
Cerebrovascular disease	14.9	39	5.3	26	11.3
Cognitive dysfunction	42	0	11.6	NA	17
Demyelinating syndrome	1.8	3.1	NA	NA	1.8
Headache	78.5	10.9	13	36	39.6
Movement disorders	17.7	0	3.4	16	5.6
Mood disorders	48.6	12.5	5.4	NA	9.4
Myelopathy	0.9	4.7	NA	NA	NA
Pychosis	9.3	21.9	2.1	13	9.4
Seizures	19.6	84.4	9.5	50	35.8
Acute inflammatory demyelinating polyradiculoneuropathy	1	0	NA	6	NA
Autonomic disorders	2.8	1.6	NA	NA	NA
Cranial neuropathy	5.6	1.6	0.7	NA	1
Mononeuropathy	8.4	3.1	NA	NA	NA
Myastenia gravis	0	0	NA	NA	NA
Plexopathy	0.9	0	NA	NA	NA
Polyneuropathy	1.8	4.7	N	NA	NA

Seventeen of 107 NP-SLE patients (15.8%) experienced one neurological manifestation (other than isolated headaches, mood disorder, and/or anxiety), while 15/107 (14%) experienced two, 25/107 (23.3%) experienced three, 21/107 (19.6%) experienced four; and the remaining 29/107 subjects (27%) had five or more concurrent NP manifestations. There were no significant differences in the presence of NP events based on age at diagnosis (mean age 12.2 years for patients without NP involvement vs 11.5 years in patients with NP involvement, *p* = 0.05).

### Laboratory features associated with NP-JSLE

Between JSLE patients with versus without NP involvement, no statistically significant differences were seen in relation to autoantibody positivity (ANA, Anti-Sm, anti-dsDNA, anti-SS-A/Ro and SS-B/La, anti-phospholipid), white blood cell counts (total, neutrophils, lymphocytes), erythrocyte sedimentation rate (ESR), and levels of hemoglobin, serum creatinine, thyroid-stimulating hormone, thyroxine, total-, LDL-, and HDL-cholesterol.

**Figure 2.**
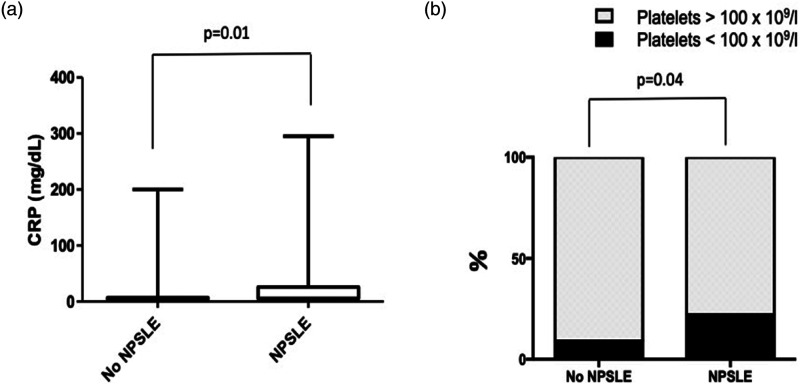
Significant differences in laboratory findings in juvenile-onset systemic lupus erythematosus patients with or without neuropsychiatric involvement. Neuropsychiatric, NP; Systemic lupus erythematosus, SLE; CRP, C-reactive protein. Low platelet numbers <100 × 10^9^/L. For continuous variables, Mann–Whitney *U* test was used; for categorical variables, Chi-square test was used.

Significantly reduced platelet counts (<100 × 10^9^/L) were observed at first visit in patients with NP involvement (*p* = 0.04); serum C-reactive protein (CRP) levels were higher in patients with NP involvement (mean: 28.2 mg/L in children with NP features, vs 11.7 mg/L in those without; *p* = 0.01) (Figure 2).

### Disease activity and damage

NP involvement was associated with an increased number of ACR criteria fulfilled at last visit (*p* < 0.001) (Table 3). The total pBILAG numerical disease activity score at first (*p* < 0.001) visit, and SLICC damage scores at first (*p* = 0.02) and last (*p* < 0.001) visit were significantly higher in patients with NP involvement. In JSLE patients without NP involvement, renal involvement predominantly affected SLICC damage scores, followed by musculoskeletal, and skin manifestations (alopecia), while in patients with NP-SLE the presence of NP disease led to the increase in SLICC scores.

**Table 3.** Disease activity and damage in JSLE with a neuropsychiatric involvement. For ACR score, mean, and range are shown for total JSLE cohort, mean (SD) are shown for subgroups to be statistically analyzed. ACR scores are counted up to the time of that visit.

	All JSLE patients	JSLE patients with NP involvement	JSLE patients without NP involvement	*p value*	JSLE patients with early NP involvement	JSLE patients with late NP involvement	*p value*
ACR score at first visit	4.7 (2–9)	4.9 (1.5)	4.6 (1.2)	0.07	5 (1.5)	4.8 (1.4)	0.34
ACR score at last visit	5.6 (4–10)	6 (3.3)	5.5 (1.3)	**<0.001**	6.3 (1.8)	5.8 (1.4)	0.09
Global BILAG score (mean) at first visi	10.2	13.4	9.2	**<0.001**	15.2	11.7	0.1
Global BILAG score (mean) at last visit	2.7	3.1	2.6	0.27	3.8	2.5	0.1
SLICC damage score (mean) at first visit	0.2	0.3	0.2	**0.02**	0.6	0	**0.01**
SLICC damage score (mean) at last	0.6	1.2	0.4	**<0.001**	1.7	0.7	**0.009**

ACR: American College of Rheumatology; BILAG: British Isles Lupus Assessment Grade; SLICC: Systemic Lupus International Collaborating Clinic damage index; NP: neuropsychiatric; JSLEs: juvenile systemic lupus erythematosus. ACR, global pBILAG2004, and SLICC damage index scores at the first and last visit, distinguishing JSLE patients with and without NP involvement, as well as those with NP involvement already at first visit (“early” NP) respect to those that develop it later (“late” NP). SD, standard deviation. For continuous variables, Mann–Whitney *U* test was used; for categorical variables, Chi-square test was used.

38% (20/52) of patients with “early” NP involvement had “severe” NP involvement, 56.4% (29/52) had “moderate” NP involvement, and the remaining 5.1% (3/52) of patients a “mild” NP involvement. At last visit, 6/52 (11.5%) patient with “early” NP involvement still had a “moderate” pBILAG defined NP involvement, while the majority of patients (46/52, 88.4%) had inactive neurological disease.

Among those 55 patients with a “late” NP involvement (namely, developed after their first visit), 2/55 (3.6%) had a “severe” NP pBILAG score at last visit, 7/55 (12.7%) had a “moderate” score, 5/55 (9%) a “mild” score, and 28/55 (51%) were inactive at last visit.

Five (1.1%) JSLE patients included in this study died during the observation period, two of whom experienced NP involvement; the small numbers limit statistical considerations.

## Discussion

The current study is the largest to date examining the prevalence and associations with NP-SLE in a national JSLE population. Despite a conservative case definition for NP-SLE (in which isolated headaches, mood disorder or anxiety were not included if not accompanied by additional NP signs or symptoms), NP involvement was seen in 25% of UK JSLE Cohort Study patients within the first 5 years from diagnosis. NP involvement was generally shown to occur independently of single clinical and/or serologic SLE disease activity markers. The only laboratory parameters associated were low platelets and high CRP. N*p* involvement however was associated with additional SLE features (ACR classification criteria defined), and with higher global disease activity and damage scores.

NP involvement can be the result of antibody-mediated pathology, vasculitis, and/or increased permeability and dysfunction of the blood–brain/CSF barrier, resulting in the influx of neuropathic antibodies, cytokines, and lymphocytes promoting inflammation and damage.^20^ The situation is further complicated by chronic systemic inflammation, impairment/damage to other organs, and/or treatment side-effects potentially contributing to NP manifestations (e.g. mood disorders and/or headaches). Though the pathophysiological causes are largely unknown, increased incidence, and severity of NP involvement in JSLE as compared to adult-onset SLE patients may be linked to a higher prevalence of rare “monogenic” disease causes,^1,9,10,11,21–23^ and overall increased genetic burden among JSLE patients.^1,11,24^ Furthermore, immunological, neuro-anatomical, and developmental differences between children and adults may play a role.^21^

Depending on the study, patient cohorts included, and case definitions applied, NP manifestations have been reported in 14%–80% of adult-onset SLE patients, and in 22%–95% of JSLE patients.^7,25–32^ Stringent criteria were applied in the current study (as outlined above), excluding patients with singular potentially unspecific symptoms. Consequently, 25% of JSLE patients were classed as having NP involvement, with nearly half of NP-JSLE patients exhibiting NP involvement already at the time of their first hospital visit.

In a cohort of 185 Chinese JSLE patients, 22% of NP-SLE patients had NP symptoms at diagnosis, and 32.8% within the first year.^32^ Among 146 Iranian JSLE patients, as many as 43.9% exhibited NP disease at the time of diagnosis, an additional 24.4% developed it during their first year.^33^ The higher incidences of NP involvement reported in these studies may relate to the case definitions used, in particular, not excluding patients with isolated headaches from the study population.

In agreement with other studies,^32–35^ we observed a female predominance (female:male ratio of 5.4:1), and a median age at diagnosis of 12.2 years in both entire cohort and the NP-JSLE sub-population. Similarly, NP-SLE patients more frequently experienced CNS (as compared to PNS) involvement, which agrees with the published literature.^6,7,12,25,30,32–35^ However, the distribution of individual neurological symptoms differs between studies, with increased prevalence of headaches, cognitive impairment, mood disorders, and anxiety, and a lower frequency of seizures in the this study population.^32–35^ Notably, while ethnic minority groups were over-represented among JSLE patients as compared to national census data from the UK, sex, and racial composition did not differ between JSLE patients with vs patients without NP involvement. Of note, one of the particular strengths of this study is the multiethnic composition of the UK JSLE Cohort Study, as other studies were performed in relatively homogenous populations.^32–35^

As reported previously by others, headaches were the most common NP symptom in the UK JSLE Cohort (78.5%), in accordance with adult SLE series reporting prevalence of headaches to range between 23% and 68%.^26,27^ The ACR case definition of NP syndromes mentions five types of headaches (migraine, tension headache, cluster headache, headache due to intracranial hypertension, and intractable nonspecific headache) based on the International Headache Society classification, without distinctions indicative for specific SLE etiology.^8,36^ All of these are reflected in the UK JSLE Cohort with similar distribution between the NP-SLE group and the remaining JSLE patient sub-cohort.

Psychiatric symptoms were the second most common group of NP manifestations in this cohort. On the basis of the *Diagnostic and Statistical Manual of Mental Disorders* (DSM-5), mood disorders encompass two main groups of conditions: depressive and related disorders, and bipolar, and related disorders. Usually, SLE is associated with depressive symptoms, while bipolar disorders are rare.^37^ In this study, almost 48% of NP-JSLE patients exhibited mood disorder. In 17.3% of the cases, symptoms were severe and classified as “major depression.” While mood disorders can generally occur in patients with chronic disease, previous studies have suggested that anxiety levels are significantly higher in adult-onset SLE patients when compared to healthy controls or RA patients (*p* = 0.02 and 0.01, respectively).^38^ Severe anxiety was present in 23.3% of NP-SLE patients in the current study, while an acute confusional state was observed in a small percentage of children (6.5% of NP-SLE patients).

Psychosis was observed in 10 patients (9.3%), six with an acute manifestation and four with a chronic course. Psychosis is a rarely reported feature, mainly during the early phases of the disease course; it was present in 28/1826 (1.53%) of adult SLE patients, mostly occurring early after disease onset and associating with a negative impact on health status.^39^

This and data from additional pediatric studies (Table 2) suggests markedly higher prevalence of psychosis in JSLE as compared to adult-onset SLE patients.^32-34^ Among environmental or social factors, differences between studies may be explained by ethnicity-related genetic variables that affects both the disease’s clinical course and outcomes in JSLE. Indeed, considering Asian JSLE populations, in the UK, East Asian patients are under-represented, while South Asian patients account for the majority of Asian JSLE patients.^2^

Approximately 15% of the entire UK JSLE Cohort experienced cognitive impairment. The majority (69%) already exhibited this at first visit. Due to its variable presentation, and the difficulty verifying/quantifying cognitive impairment, the reported incidence of cognitive impairment in adult-onset SLE cohorts has been shown to vary widely (6–80%).^40^ Similar variability is also observed in JSLE (see Table 2) with neurocognitive impairment reported in up to 71% of US American JSLE patients^41^ and the University of New Mexico Lupus Cohort^25^ demonstrating cognitive disorder in 55% of JSLE cases. Again, differences in ethnicity may in part account for some of the variability between published cohorts. Cognitive dysfunction can also include a wide range of symptoms, affecting language, attention, reasoning, memory, executive skills, visual-spatial processing and psychomotor skills, accounting for some of these differences.^8^ The presence of potential confounders, such as socioeconomic factors, infections, or drug side-effects (e.g. corticosteroids, hydroxychloroquine), the lack of standardized assessments, and limitations of available neuropsychological tests contribute to under-diagnosis and imprecise estimates of the prevalence, incidence, and severity of cognitive impairment. This is further complicated in childhood where a degree of developmental variability is physiological. Lastly, overlapping mood disturbances can mask or complicate cognitive disorders. Indeed, 50% of the UK JSLE Cohort patients experiencing cognitive dysfunction had a concurrent mood disorder, such as depression or psychosis.

In this study, 19.6% of NP-SLE patients experienced seizures, mostly during the early phase of the disease (17/21). In two patients (1.8%), a diagnosis of epilepsy was made. Compared to other published reports, this proportion is relatively low (see Table 2). In adult-onset SLE cohorts, epilepsy, and/or seizures occur in up to 11.5% of patients.^42^ Younger patient age and high disease activity may be independent predictors of seizures.^43^ In a Chinese cohort of juvenile NP-SLE patients, seizures were one of the most frequent NP-JSLE–related symptoms, observed in 84% of NP-JSLE patients.^32^ Of note, half of patients enrolled in the UK JSLE Cohort Study who developed seizures were Caucasian and half South Asian. As 53.2% of NP-SLE patients were of White Caucasian, and 24.4% of South Asian descent, this suggests an over-representation of South Asian patients in this sub-cohort. Seizures are associated with the presence of anti-phospholipid (aPL)^44^ and anti-β2 glycoprotein (GPI) antibodies^45^ in adult-onset SLE patients, with GPI antibodies linked to intractable headaches and ischemic stroke. Incomplete data on the presence of aPL antibodies and a lack of centralized aPL testing precludes meaningful assessment of the role of such antibodies in the current cohort.

Cerebrovascular disease affected 14.9% of NP-JSLE patients in this study, in 87% at disease onset. Ischemic stroke accounted for half of cerebrovascular events, in contrast to 5–39% in reported cases.^32,33^ However, some studies did not distinguish stroke within this category by combining CNS vasculitis as well. In patients with adult-onset SLE the risk of ischemic stroke is two-fold higher when compared to the general population,^46^ and younger subjects (<50 years of age) in an early and active phase of the disease are more prone to develop this complication.^47^ Socioeconomic and racial variations may influence the risk for CNS infarctions, with Black and Hispanic patients being at a particular high risk.^48^ In this study, cerebrovascular involvement (including vasculitis and stroke) in 50% affected white Caucasian and in 50% South Asian patients, thus over-representing South Asians (24.2% of NP-SLE sub-cohort). Lastly, aPL antibody positivity increases the stroke risk at any time during the disease course, independent of the inflammatory activity.^47^ As mentioned above, aPL were not determined in all cases here which makes an assessment impossible.

Movement disorders, including chorea, cerebellar ataxia, and other types of abnormal movements, were present in 17.7% of the presented NP-JSLE cohort. This is comparable to the reported frequency in a Columbian juvenile NP-SLE population, while movement disorders were less common in Indian, Iranian, and in the Chinese cohort in which no patient showed this complication.^32–35^

While most patients experienced CNS symptoms related to NP-SLE, 7% developed peripheral neuropathy. Within this group, mononeuropathy (41%), cranial nerve involvement (27%), polyneuropathy (9%), and autonomic neuropathy (13.6%) were most common. In the adult literature, peripheral involvement has been reported in 2.2–32%, and appears to correlate with disease activity.^49^ Limited reports in the pediatric age group suggest PNS involvement in 0.7–13%.^50^ The small number of patients with PNS involvement in the current cohort prevents any meaningful analysis.

Comparing JSLE patients with NP involvement to the rest of the UK JSLE Cohort, no differences were recorded in autoantibody patterns and immune cell counts. Low platelet counts (<100 × 10^9^/L) were more common in NP-SLE patients at first visit. Furthermore, CRP was higher in NP-SLE patients. Of note, associations between CRP elevation and disease activity in SLE have been established.^51^ However, in suspected cases of NP-SLE it is crucial that infections (in particular meningitis and encephalitis) are considered and excluded. NP-SLE patients also exhibit a higher total numerical pBILAG disease activity scores at first visit, and an overall higher number of ACR criteria present at last visit, with higher SLICC damage index scores at first and last visit. Lastly, among patients with NP-JSLE, those with neurologic involvement early in the disease course exhibited higher disease activity (pBILAG) when compared to those with a later onset of NP symptoms. Taken together, laboratory, and clinical data from this study suggest a relationship between high disease activity and the development of NP involvement. This, though numbers are too low to draw definite conclusions, is underpinned by increased mortality in NP-SLE patients from the UK JSLE Cohort; five deaths were recorded in the entire UK JSLE cohort (mortality overall: 1.1%), two in the NP-JSLE sub-group (mortality: non–NP-SLE: 0.7% vs NP-SLE 1.86%).

We acknowledge certain strengths and limitations of this study. This is the one of the largest studies to date offering a detailed description of the different features related to NP-SLE. In contrast to other recent large NP-JSLE case series, which included Middle Eastern, South Asian, and East Asian patients, this multiethnic cohort also includes Black African/Caribbean and White Caucasian patients. Limitations include incomplete datasets in relation to autoantibody status (in particular the presence of aPL was not always reported), and the possible underestimation of mild NP signs or symptoms. Furthermore, exclusion of patients with singular headaches, mood disorder, or anxiety may have resulted in an underestimation of NP-JSLE prevalence and incidence, but limits false inclusion of individuals, for example, experiencing drug-related symptoms (e.g. dysphoria in the context of corticosteroids).

## Conclusions

NP involvement is common in this national cohort of JSLE patients (25%), and in half of all cases already present at diagnosis. Clinical features are variable and can be nonspecific, which makes diagnosis a challenge and holds the potential of under-recognition and the development of complications. To avoid diagnostic and therapeutic delay, NP involvement should be considered in all JSLE patients, including testing for cognitive impairment. Of note, NP-JSLE is associated with increased disease activity (pBILAG, and CRP elevation), and damage (SLICC-SDI index) that may contribute to increased mortality. Future studies are warranted, focusing upon the impact of NP involvement on treatment response, permanent damage, and quality of life. International collaboration is needed to dissect disease aspects related to ethnic, genetic, and socioeconomic factors.
